# Carbonic anhydrase I, II, IV and IX inhibition with a series of 7-amino-3,4-dihydroquinolin-2(1H)-one derivatives

**DOI:** 10.1080/14756366.2017.1337759

**Published:** 2017-06-23

**Authors:** Murat Bozdag, Silvia Bua, Sameh M. Osman, Zeid AlOthman, Claudiu T. Supuran

**Affiliations:** a Dipartimento di Chimica e Dipartimento Neurofarba, Sezione di Scienze Farmaceutiche e Nutraceutiche, Università degli Studi di Firenze, Florence, Italy;; b Department of Chemistry, College of Science, King Saud University, Riyadh, Saudi Arabia

**Keywords:** Carbonic anhydrase, inhibitor, coumarin, dihydroquinolinone, sulfonamide

## Abstract

A series of new derivatives was prepared by derivatisation of the 7-amino moiety present in 7-amino-3,4-dihydroquinolin-2(1H)-one, a compound investigated earlier as CAI. The derivatisation was achieved by: i) reaction with arylsulfonyl isocyanates/aryl isocyanates; (ii) reaction with fluorescein isothiocyanate; (iii) condensation with substituted benzoic acids in the presence of carbodiimides; (iv) reaction with 2,4,6-trimethyl-pyrylium tetrafluoroborate; (v) reaction with methylsulfonyl chloride and (vi) reaction with maleic anhydride. The new compounds were assayed as inhibitors of four carbonic anhydrases (CA, EC 4.2.1.1) human (h) isoforms of pharmacologic relevance, the cytosolic hCA I and II, the membrane-anchored hCA IV and the transmembrane, tumour-associated hCA IX. hCA IX was the most inhibited isoform (*K_I_*s ranging between 243.6 and 2785.6 nm) whereas hCA IV was not inhibited by these compounds. Most derivatives were weak hCA I and II inhibitors, with few of them showing *K_I_*s < 10 µm. Considering that the inhibition mechanism with these lactams is not yet elucidated, exploring a range of such derivatives with various substitution patterns may be useful to identify leads showing isoform selectivity or the desired pharmacologic action.

## Introduction

CO_2_, bicarbonate and protons are essential molecules/ions in important physiologic processes in the three life kingdoms (*Bacteria*, *Archaea* and *Eukarya*), and for this reason, relatively high amounts of the enzymes carbonic anhydrases (CAs, EC 4.2.1.1) are present in different tissues/cell compartments of most investigated organisms^1–11^. The α-CAs are present in vertebrates, protozoa, algae and cytoplasm of green plants and in some *Bacteria*^1–19^, the β-CAs are predominantly found in *Bacteria*, algae and chloroplasts of both mono- as well as dicotyledons, but also in many fungi and some *Archaea*^1–11^. The γ-CAs were found in plants, *Archaea* and *Bacteria*^1–11^, whereas the δ-, ζ- and θ-CAs seem to be present only in marine diatoms^11^. The η-CA class has been discovered in protozoa such as those belonging to the genus *Plasmodium*^20^. In many organisms, these enzymes are involved in crucial physiological processes connected with respiration and transport of CO_2_/bicarbonate, pH and CO_2_ homeostasis, electrolyte secretion in a variety of tissues/organs, biosynthetic reactions (e.g. gluconeogenesis, lipogenesis and ureagenesis), bone resorption, calcification, tumourigenicity and many other physiologic or pathologic processes (thoroughly studied in vertebrates)^1–11^^,^^21–26^, whereas in algae, plants and some bacteria they play an important role in photosynthesis and other biosynthetic reactions^8^^,^^11^. In diatoms δ- and ζ-CAs play a crucial role in carbon dioxide fixation^11^. Many such enzymes from vertebrates, fungi and bacteria are well-known drug targets, with inhibitors and activators possessing various pharmacologic applications^23–42^.

Sulfonamides are the most important class of CA inhibitors CAIs^1^^,^^4–12^, with several compounds in clinical use for many years, as diuretics^1^^,^^26^^,^^28^, antiglaucoma agents^1^^,^^27^^,^^33^, antiepileptics^30–34^ and more recently as anticancer agents^1^^,^^2^^,^^12^. Although a large number of isoform-selective sulfonamide CAIs were reported ultimately, mostly by using the tail approach for their synthesis^16–23^^,^^26^, a large variety of other chemotypes were investigated for their interaction with these enzymes, which led to the development of a large number of non-classic CAIs, belonging to various classes^14^^,^^33^. Here, we report a new series of such derivatives which incorporate the 7-amino-3,4-dihydroquinolin-2(1H)-one scaffold^43^.

## Materials and methods

### Chemistry

Anhydrous solvents and all reagents were purchased from Sigma-Aldrich (Milan, Italy). All reactions involving air- or moisture-sensitive compounds were performed under a nitrogen atmosphere using dried glassware and syringes techniques to transfer solutions. Nuclear magnetic resonance (^1^H-NMR, ^13^C-NMR) spectra were recorded using a Bruker Advance III 400 MHz spectrometer in DMSO-d_6_. Chemical shifts are reported in parts per million (ppm) and the coupling constants (*J*) are expressed in Hertz (Hz). Splitting patterns are designated as follows: s, singlet; d, doublet; t, triplet; q, quadruplet; dd, double of doublet. The assignment of exchangeable protons (O*H* and N*H*) was confirmed by the addition of D_2_O.

### General procedure for the preparation of compounds 2–20

A solution of 7-amino-3,4-dihydroquinolin-2(1H)-one (**1**) in dry dimethylformamide (3–5 ml) was treated with a stoichiometric amount of appropriate isocyanates/isothiocyanate. The mixture was stirred at room temperature until the consumption of starting materials (TLC monitoring). The reaction was quenched with a 1.0 M aqueous solution of HCl to give a precipitate that was washed with diethyl ether (3 × 5 ml), filtered and dried under vacuum (compounds 2–19) or extracted with ethyl acetate (3 × 15 ml), the combined organic layers were washed with H_2_O (3 × 15 ml), dried over Na_2_SO_4_, filtered, and concentrated (compound 20) to afford the title compounds **2–20**.

### N-((2-oxo-1,2,3,4-tetrahydroquinolin-7-yl)carbamoyl)benzenesulfonamide (2)

Beige solid, yield 89%; m.p.: 272–273 °C; silica gel TLC *R_f_* = 0.16 (MeOH/DCM 10% *v/v*); *δ*_H_ (400 MHz, DMSO-d_6_) 2.42 (2H, d, *J* 6.8), 2.81 (2H, d, *J* 6.8), 6.84 (1H, dd, *J* 2.0, 8.4), 7.02 (1H, d, *J* 2.0), 7.06 (1H, d, *J* 8.4), 7.67 (2H, t, *J* 8.0), 7.73 (1H, t, *J* 8.0), 8.00 (2H, d, *J* 8.0), 8.91 (1H, s, exchange with D_2_O, N*H*), 10.03 (1H, s, exchange with D_2_O, N*H*), 10.70 (1H, s, exchange with D_2_O, N*H*); *δ*_C_ (100 MHz, DMSO-d_6_) 25.2, 31.7, 106.2, 112.9, 117.2, 127.7, 128.3, 129.1, 131.9, 139.2, 140.4, 145.2, 171.2; *m/z* (ESI negative) 344.0 [M − H]^−^.

### 4-Methyl-N-((2-oxo-1,2,3,4-tetrahydroquinolin-7-yl)carbamoyl)benzenesulfonamide (3)

White solid, yield 60%; m.p.: 260–261 °C; silica gel TLC *R_f_* = 0.16 (Ethyl acetate 100% *v/v*); *δ*_H_ (400 MHz, DMSO-d_6_) 2.43 (5H, m), 2.81 (2H, t, *J* 7.8), 6.84 (1H, dd, *J* 2.0, 8.0), 7.01 (1H, d, *J* 2.0), 7.06 (1H, d, *J* 8.0), 7.46 (2H, d, *J* 8.4), 7.87 (2H, d, *J* 8.4), 8.82 (1H, s, exchange with D_2_O, N*H*), 10.03 (1H, s, exchange with D_2_O, N*H*); *δ*_C_ (100 MHz, DMSO-d_6_) 22.0, 25.1, 31.5, 106.8, 113.4, 119.3, 128.4, 128.8, 130.4, 137.9, 138.1, 139.5, 144.8, 150.1, 171.1; *m/z* (ESI negative) 358.0 [M − H]^−^.

### 2-Methyl-N-((2-oxo-1,2,3,4-tetrahydroquinolin-7-yl)carbamoyl)benzenesulfonamide (4)

White solid, yield 79%, m.p.: 285–286 °C; silica gel TLC *R_f_* = 0.43 (Ethyl acetate 100% *v/v*); *δ*_H_ (400 MHz, DMSO-d_6_) 2.42 (2H, d, *J* 7.6), 2.66 (3H, s), 2.80 (2H, t, *J* 7.6), 6.81 (1H, d, *J* 8.0), 7.05 (2H, m), 7.47 (2H, m), 7.61 (1H, m), 8.01 (1H, d, *J* 7.6), 8.69 (1H, s, exchange with D_2_O, N*H*), 10.02 (1H, s, exchange with D_2_O, N*H*), 10.58 (1H, s, exchange with D_2_O, N*H*); *δ*_C_ (100 MHz, DMSO-d_6_) 20.6, 25.1, 31.5, 106.7, 113.3, 119.3, 127.2, 128.8, 131.0, 133.3, 134.3, 137.6, 137.8, 138.8, 139.6, 149.8, 171.1; *m/z* (ESI negative) 358.0 [M − H]^−^.

### 4-Chloro-N-((2-oxo-1,2,3,4-tetrahydroquinolin-7-yl)carbamoyl)benzenesulfonamide (5)

White solid, yield 67%; m.p.: 253–254 °C; silica gel TLC *R_f_* = 0.35 (MeOH/DCM 10% *v/v*); *δ*_H_ (400 MHz, DMSO-d_6_) 2.43 (2H, t, *J* 6.8), 2.81 (2H, t, *J* 6.8), 6.85 (1H, dd, *J* 2.0, 8.4), 7.01 (1H, d, *J* 2.0), 7.06 (1H, d, *J* 8.4), 7.75 (2H, d, *J* 8.8), 8.01 (2H, d, *J* 8.8), 8.94 (1H, s, exchange with D_2_O, N*H*), 10.03 (1H, s, exchange with D_2_O, N*H*), 10.81 (1H, s, exchange with D_2_O, N*H*); *δ*_C_ (100 MHz, DMSO-d_6_) 25.1, 31.5, 107.0, 113.5, 119.5, 128.8, 130.1, 130.4, 137.8, 139.2, 139.6, 139.8, 150.1, 171.1; *m/z* (ESI negative) 378.0 [M − H]^−^.

### 4-Fluoro-N-((2-oxo-1,2,3,4-tetrahydroquinolin-7-yl)carbamoyl)benzenesulfonamide (6)

White solid, yield 68%; m.p.: 245–246 °C; silica gel TLC *R_f_* = 0.23 (Ethyl acetate/*n*-hexane 80% *v/v*); *δ*_H_ (400 MHz, DMSO-d_6_) 2.42 (2H, t, *J* 7.6), 2.81 (2H, t, *J* 7.6), 6.85 (1H, dd, *J* 1.8, 8.1), 7.02 (1H, d, *J* 1.8), 7.06 (1H, d, *J* 8.1), 7.51 (2H, m), 8.06 (2H, m), 8.92 (1H, s, exchange with D_2_O, N*H*), 10.04 (1H, s, exchange with D_2_O, N*H*), 10.77 (1H, brs, exchange with D_2_O, N*H*); *δ*_C_ (100 MHz, DMSO-d_6_) 25.1, 31.5, 106.9, 113.4, 117.2 (d, ^2^*J*_C–F_ 23), 119.4, 128.8, 131.6 (d, ^3^*J*_C–F_ 10), 137.2 (d, ^4^*J*_C–F_ 3), 137.8, 139.5, 150.1, 165.6 (d, ^1^*J*_C–F_ 250), 171.1; *δ*_F_ (376 MHz, DMSO-d_6_) −105.1 (1 F, s); *m/z* (ESI negative) 362.0 [M − H]^−^.

### 1-(2-Oxo-1,2,3,4-tetrahydroquinolin-7-yl)-3-phenylurea (7)

White solid, yield 85%; m.p.: 255–256 °C (dec.); silica gel TLC *R_f_* = 0.65 (MeOH/DCM 10% *v/v*); *δ*_H_ (400 MHz, DMSO-d_6_) 2.46 (2H, d, *J* 7.6), 2.83 (2H, d, *J* 7.6), 6.99 (2H, m), 7.08 (2H, m), 7.31 (2H, d, *J* 7.9), 7.47 (2H, d, *J* 7.9), 8.60 (1H, s, exchange with D_2_O, N*H*), 8.66 (1H, s, exchange with D_2_O, N*H*), 10.09 (1H, s, exchange with D_2_O, N*H*); *δ*_C_ (100 MHz, DMSO-d_6_) 25.2, 31.7, 106.1, 112.7, 117.9, 119.0, 122.7, 128.8, 129.7, 139.5, 139.6, 140.6, 153.3, 171.2; *m/z* (ESI positive) 282.0 [M + H]^+^.

### 1-(2-Oxo-1,2,3,4-tetrahydroquinolin-7-yl)-3-(p-tolyl)urea (8)

White solid, yield 88%; m.p.: 276–277 °C; silica gel TLC *R_f_* = 0.48 (MeOH/DCM 10% *v/v*); *δ*_H_ (400 MHz, DMSO-d_6_) 2.28 (3H, s), 2.46 (2H, t, *J* 7.6), 2.83 (2H, t, *J* 7.6), 7.00 (1H, dd, *J* 2.0, 8.4), 7.09 (4H, m), 7.35 (2H, d, *J* 8.4), 8.48 (1H, s, exchange with D_2_O, N*H*), 8.60 (1H, s, exchange with D_2_O, N*H*), 10.07 (1H, s, exchange with D_2_O, N*H*); *δ*_C_ (100 MHz, DMSO-d_6_) 21.2, 25.1, 31.6, 106.0, 112.6, 117.7, 119.1, 128.7, 130.0, 131.5, 138.0, 139.5, 139.7, 153.3, 171.2; *m/z* (ESI positive) 296.0 [M + H]^+^.

### 1-(2-Oxo-1,2,3,4-tetrahydroquinolin-7-yl)-3-(o-tolyl)urea (9)

White solid, yield 90%; m.p.: > 300 °C; silica gel TLC *R_f_* = 0.47 (MeOH/DCM 10% *v/v*); *δ*_H_ (400 MHz, DMSO-d_6_) 2.27 (3H, s), 2.46 (2H, t, *J* 6.8), 2.83 (2H, t, *J* 6.8), 6.97 (1H, t, *J* 7.2), 7.07 (3H, m), 7.18 (2H, m), 7.89 (2H, m, 1H exchange with D_2_O, N*H*), 9.01 (1H exchange with D_2_O, N*H*), 10.11 (1H exchange with D_2_O, N*H*); *δ*_C_ (100 MHz, DMSO-d_6_) 18.8, 25.1, 31.7, 105.9, 112.5, 117.7, 121.6, 123.4, 127.1, 128.1, 128.8, 131.1, 138.4, 139.5, 139.8, 153.4, 171.2; *m/z* (ESI positive) 296.0 [M + H]^+^.

### 1-(4-Chlorophenyl)-3-(2-oxo-1,2,3,4-tetrahydroquinolin-7-yl)urea (10)

White solid, yield 97%; m.p.: 249–250 °C; silica gel TLC *R_f_* = 0.55 (Ethyl acetate 100% *v/v*); *δ*_H_ (400 MHz, DMSO-d_6_) 2.46 (2H, t, *J* 7.6), 2.83 (2H, t, *J* 7.6), 7.00 (1H, dd, *J* 2.0, 8.4), 7.08 (2H, m), 7.35 (2H, d, *J* 9.2), 7.50 (2H, d, *J* 9.2), 8.08 (1H, s, exchange with D_2_O, N*H*), 8.88 (1H, s, exchange with D_2_O, N*H*), 10.09 (1H, s, exchange with D_2_O, N*H*); *δ*_C_ (100 MHz, DMSO-d_6_) 25.2, 31.7, 106.2, 112.8, 118.0, 120.5, 126.1, 128.8, 129.5, 139.5, 139.5, 139.7, 153.3, 171.2; *m/z* (ESI positive) 316.0 [M + H]^+^.

### 1-(2-Chlorophenyl)-3-(2-oxo-1,2,3,4-tetrahydroquinolin-7-yl)urea (11)

White solid, yield 83%; m.p.: > 300 °C; silica gel TLC *R_f_* = 0.50 (Ethyl acetate 100% *v/v*); *δ*_H_ (400 MHz, DMSO-d_6_) 2.46 (2H, d, *J* 7.2), 2.84 (2H, t, *J* 7.2), 7.08 (4H, m), 7.33 (1H, t, *J* 8.0), 7.49 (1H, d, *J* 8.0), 8.20 (1H, d, *J* 8.0), 8.30 (1H, s, exchange with D_2_O, N*H*), 9.41 (1H, s, exchange with D_2_O, N*H*), 10.14 (1H, s, exchange with D_2_O, N*H*); *δ*_C_ (100 MHz, DMSO-d_6_) 25.1, 31.6, 106.0, 112.6, 118.2, 122.0, 122.7, 124.1, 128.5, 128.9, 130.1, 136.9, 139.3, 139.6, 152.9, 171.2; *m/z* (ESI positive) 316.0 [M + H]^+^.

### 1-(4-Fluorophenyl)-3-(2-oxo-1,2,3,4-tetrahydroquinolin-7-yl)urea (12)

White solid, yield 98%; m.p.: 257–258 °C; silica gel TLC *R_f_* = 0.59 (Ethyl acetate 100% *v/v*); *δ*_H_ (400 MHz, DMSO-d_6_) 2.45 (2H, t, *J* 7.8), 2.83 (2H, t, *J* 7.8), 7.00 (1H, dd, *J* 2.0, 8.8) 7.08 (2H, m), 7.14 (2H, m), 7.48 (2H, m), 8.62 (1H, s, exchange with D_2_O, N*H*), 8.64 (1H, s, exchange with D_2_O, N*H*), 10.08 (1H, s, exchange with D_2_O, N*H*); *δ*_C_ (100 MHz, DMSO-d_6_) 25.2, 31.6, 106.2, 112.8, 116.1 (d, ^2^*J*_C–F_ 22), 117.9, 120.7 (d, ^3^*J*_C–F_ 8), 128.8, 137.0 (q, *J*_C–F_ 2), 139.5, 139.6, 153.4, 158.5 (d, ^1^*J*_C–F_ 237), 171.2; *δ*_F_ (376 MHz, DMSO-d_6_) −121.5 (1 F, s); *m/z* (ESI positive) 300.0 [M + H]^+^.

### 1-(4-Fluoro-3-methylphenyl)-3-(2-oxo-1,2,3,4-tetrahydroquinolin-7-yl)urea (13)

White solid, yield 89%; m.p.: > 300 °C; silica gel TLC *R_f_* = 0.47 (MeOH/DCM 10% *v/v*); *δ*_H_ (400 MHz, DMSO-d_6_) 2.24 (3H, d, *J* 1.5), 2.45 (2H, t, *J* 7.6), 2.82 (2H, t, *J* 7.6), 7.00 (1H, dd, *J* 2.0, 8.10), 7.07 (3H, m), 7.27 (1H, m), 7.38 (1H, m), 8.55 (1H, exchange with D_2_O, N*H*), 8.64 (1H, s, exchange with D_2_O, N*H*), 10.07 (1H, s, exchange with D_2_O, N*H*); *δ*_C_ (100 MHz, DMSO-d_6_) 15.3 (d, *J*_C–F_ 3), 25.2, 31.7, 106.2, 112.8, 115.8 (d, ^2^*J*_C–F_ 23), 117.9, 118.2 (d, ^3^*J*_C–F_ 8), 122.1 (d, ^3^*J*_C–F_ 4), 125.1 (d, ^2^*J*_C–F_ 18), 128.8, 136.6 (d, ^4^*J*_C–F_ 3), 139.5, 139.7, 153.5, 157.0 (d, *J*_C–F_ 236), 171.3; *δ*_F_ (376 MHz, DMSO-d_6_) −125.9 (1 F, s); *m/z* (ESI positive) 314.0 [M + H]^+^.

### 1-(2,4-Difluorophenyl)-3-(2-oxo-1,2,3,4-tetrahydroquinolin-7-yl)urea (14)

White solid, yield 95%; m.p.: 240–241 °C; silica gel TLC *R_f_* = 0.42 (MeOH/DCM 10% *v/v*); *δ*_H_ (400 MHz, DMSO-d_6_) 2.46 (2H, t, *J* 7.8), 2.83 (2H, t, *J* 7.8), 7.07 (4H, m), 7.34 (1H, m), 8.13 (1H, m), 8.47 (1H, s, exchange with D_2_O, N*H*), 9.03 (1H, s, exchange with D_2_O, N*H*), 10.11 (1H, s, exchange with D_2_O, N*H*); *δ*_C_ (100 MHz, DMSO-d_6_) 25.2, 31.7, 104.7 (t, ^2^*J*_C–F_ 24), 106.0, 111.9 (dd, ^2^*J*_C–F_ 4, 22), 112.6, 118.1, 122.7, (dd, ^3^*J*_C–F_ 3.0, 9.0), 125.1 (dd, ^3^*J*_C–F_ 3.0, 10.0), 128.9, 139.4, 139.6, 153.1 (dd, ^1^*J*_C–F_ 12.0, 244.0), 153.2, 157.7 (dd, ^1^*J*_C–F_ 12.0, 240.0), 171.3; *δ*_F_ (376 MHz, DMSO-d_6_) −124.3 (1 F, d, *J* 3.0), −118.2 (1 F, *d*, J 3.0); *m/z* (ESI positive) 318.0 [M + H]^+^.

### 1-(2-Oxo-1,2,3,4-tetrahydroquinolin-7-yl)-3-(perfluorophenyl)urea (15)

White solid, yield 88%; m.p.: 297–298 °C; silica gel TLC *R_f_* = 0.8 (Ethyl acetate 100% *v/v*); *δ*_H_ (400 MHz, DMSO-d_6_) 2.45 (2H, d, *J* 7.2), 2.83 (2H, t, *J* 7.2), 7.00 (1H, dd, *J* 2.0, 8.0), 7.09 (2H, m), 8.41 (1H, s, exchange with D_2_O, N*H*), 9.07 (1H, s, exchange with D_2_O, N*H*), 10.10 (1H, s, exchange with D_2_O, N*H*); *δ*_C_ (100 MHz, DMSO-d_6_) 25.2, 31.6, 106.5, 113.1, 115.0 (m, *J*_C–F_ 15), 118.5, 128.8, 138.1 (m, *J*_C–F_ 245), 139.1, 139.3 (m, *J*_C–F_ 245), 139.6, 143.9 (m, *J*_C–F_ 245), 152.8, 171.3; *δ*_F_ (376 MHz, DMSO-d_6_) –164.3 (1 F, t, *J* 22), −159.9 (2 F, t, *J* 23), −146.4 (2 F, d, *J* 20); *m/z* (ESI negative) 370.0 [M − H]^−^.

### 1-(2-Oxo-1,2,3,4-tetrahydroquinolin-7-yl)-3-(4-(trifluoromethyl)phenyl)urea (16)

White solid, yield 72%; m.p.: 284–285 °C; silica gel TLC *R_f_* = 0.55 (Ethyl acetate 100% *v/v*); *δ*_H_ (400 MHz, DMSO-d_6_) 2.46 (2H, t, *J* 7.6), 2.84 (2H, t, *J* 7.6), 7.02 (1H, dd, *J* 2.0, 8.0), 7.10 (2H, d, *J* 8.0), 7.67 (4H, m), 8.79 (1H, s, exchange with D_2_O, N*H*), 9.01 (1H, s, exchange with D_2_O, N*H*), 10.09 (1H, s, exchange with D_2_O, N*H*); *δ*_C_ (100 MHz, DMSO-d_6_) 25.1, 31.6, 106.3, 112.9, 118.3, 118.7, 122.6 (q, ^2^*J*_C–F_ 32), 125.4 (q, ^1^*J*_C–F_ 270), 126.9 (q, ^3^*J*_C–F_ 4), 128.8, 139.1, 139.5, 144.3 (q, ^4^*J*_C–F_ 1), 153.0, 171.1; *δ*_F_ (376 MHz, DMSO-d_6_) −60.1 (3 F, s); *m/z* (ESI positive) 350.0 [M + H]^+^.

### 1-(2-Chloro-4-(trifluoromethyl)phenyl)-3-(2-oxo-1,2,3,4-tetrahydroquinolin-7-yl)urea (17)

White solid, yield 85%; m.p.: > 300 °C; silica gel TLC *R_f_* = 0.58 (MeOH/DCM 10% *v/v*); *δ*_H_ (400 MHz, DMSO-d_6_) 2.47 (2H, t, *J* 7.2), 2.85 (2H, t, *J* 7.2), 7.10 (3H, m), 7.71 (1H, dd, *J* 1.6, 8.8), 7.91 (1H, d, *J* 1.6), 8.51 (1H, d, *J* 8.8), 8.61 (1H, s, exchange with D_2_O, N*H*), 9.61 (1H, s, exchange with D_2_O, N*H*), 10.16 (1H, s, exchange with D_2_O, N*H*); *δ*_C_ (100 MHz, DMSO-d_6_) 25.2, 31.6, 106.2, 112.8, 118.6, 121.0, 122.2, 123.7 (q, *J*_C–F_ 4), 124.6 (q *J*_C–F_ 271), 125.7 (q, *J*_C–F_ 4), 127.2 (q, *J*_C–F_ 4), 129.0, 138.9, 139.6, 140.8 (q, *J*_C–F_ 40), 152.5, 171.2; *δ*_F_ (376 MHz, DMSO-d_6_) −60.4 (3 F, s); *m/z* (ESI positive) 384.0 [M + H]^+^.

### 1-(2-Fluoro-5-(trifluoromethyl)phenyl)-3-(2-oxo-1,2,3,4-tetrahydroquinolin-7-yl)urea (18)

White solid, yield 15%; m.p.; 253–254 °C; silica gel TLC *R_f_* = 0.50 (Ethyl acetate 100% *v/v*); *δ*_H_ (400 MHz, DMSO-d_6_) 2.47 (2H, t, *J* 7.2), 2.84 (2H, t, *J* 7.2), 7.05 (1H, dd, *J* 2,8), 7.12 (2H, m), 7.42 (1H, m), 7.53 (1H, m), 8.66 (1H, m), 9.18 (1H, exchange with D_2_O, N*H*), 9.61 (1H, s, exchange with D_2_O, N*H*), 10.09 (1H, s, exchange with D_2_O, N*H*); *δ*_C_ (100 MHz, DMSO-d_6_) 25.2, 31.6, 106.1, 112.6, 117 (d, *J*_C–F_ 21), 117.3 (t, *J*_C–F_ 3), 118.5, 120.0 (m), 123.5, 126.3 (td, *J*_C–F_ 3, 32), 128.9, 129.7 (d, *J*_C–F_ 11), 138.9, 139.6, 152.9, 154.3 (d, *J*_C–F_ 248), 171.2; *δ*_F_ (376 MHz, DMSO-d_6_) −60.7 (3 F, s), −124.5 (1 F, s); *m/z* (ESI positive) 368.0 [M + H]^+^.

### 1-(3,5-Bis(trifluoromethyl)phenyl)-3-(2-oxo-1,2,3,4-tetrahydroquinolin-7-yl)urea (19)

White solid, yield 30%; m.p.; 278–279 °C; silica gel TLC *R_f_* = 0.70 (Ethyl acetate 100% *v/v*); *δ*_H_ (400 MHz, DMSO-d_6_) 2.46 (2H, t, *J* 7.5), 2.85 (2H, t, *J* 7.5), 7.05 (2H, m), 7.17 (1H, m), 7.67 (1H, s), 8.16 (2H, s), 9.00 (1H, s, exchange with D_2_O, N*H*), 9.32 (1H, s, exchange with D_2_O, N*H*), 10.08 (1H, s, exchange with D_2_O, N*H*); *δ*_C_ (100 MHz, DMSO-d_6_) 25.2, 31.6, 106.8, 113.3, 115.2 (m), 118.6, 118.8 (m), 124.2 (q, ^1^*J*_C–F_ 270), 128.8, 131.6 (q, ^2^*J*_C–F_ 32), 138.9, 139.6, 142.8, 153.2, 171.2; *δ*_F_ (376 MHz, DMSO-d_6_) −61.7 (6 F, s); *m/z* (ESI negative) 416.0 [M − H]^−^.

### 2-(6-Hydroxy-3-oxo-3H-xanthen-9-yl)-5-(3-(2-oxo-1,2,3,4-tetrahydroquinolin-7-yl)thioureido)benzoic acid (20)

Red solid, yield 75%; m.p.: 189–190 °C; silica gel TLC *R_f_* = 0.23 (MeOH/DCM 10% *v/v*); *δ*_H_ (400 MHz, DMSO-d_6_) 2.49 (2H, t, *J* 7.6), 2.89 (2H, t, *J* 7.6), 6.62 (4H, m), 6.71 (2H, d, *J* 2.0), 7.04 (2H, m), 7.18 (1H, d, *J* 8.4), 7.24 (1H, d, *J* 8.4), 7.86 (1H, dd, *J* 2.0, 8.4), 8.22 (1H, d, *J* 2.0), 10.09 (1H, s, exchange with D_2_O, N*H*), 10.12 (1H, s, exchange with D_2_O N*H*), 10.16 (2H, s, exchange with D_2_O, O*H*), 10.17 (1H, s, exchange with D_2_O, N*H*); *δ*_C_ (100 MHz, DMSO-d_6_) 25.4, 31.4, 103.2, 110.6, 111.7, 113.6, 118.4, 118.5, 121.2, 124.8, 127.4, 128.7, 130.0, 131.4, 138.9, 139.4, 142.3, 152.8, 160.5, 169.4, 171.1, 180.5; *m/z* (ESI negative) 550.0 [M − H]^−^.

### 2-((2,3-Dimethylphenyl)amino)-N-(2-oxo-1,2,3,4-tetrahydroquinolin-7-yl)benzamide (21)

A solution of **1** (1.2 mmol) was treated with mefenamic acid (2.4 mmol) in dry *N,N*-Dimethylformamide (DMF) (5 ml) then *N,N′*-Dicyclohexylcarbodiimide (DCC) (2.0 equiv.) and catalytic amount of 4-(Dimethylamino)pyridine (DMAP) were added to reaction mixture. The reaction continued until the consumption of starting materials (TLC monitoring), quenched with 1 M aqueous HCl solution and extracted with ethyl acetate (3 × 15 ml). The combined organic layers were washed with H_2_O (3 × 15 ml), dried over Na_2_SO_4_, filtered, and concentrated to obtain a residue which was purified by silica gel column chromatography eluting with ethyl acetate/*n*-hexane 50% *v*/*v* to afford titled compound.

White solid, yield 20%; m.p.: 220–221 °C; silica gel TLC *R_f_* = 0.18 (Ethyl acetate/*n*-hexane 50% *v/v*); *δ*_H_ (400 MHz, DMSO-d_6_) 2.15 (3H, s), 2.31 (3H, s), 2.48 (2H, t, *J* 7.6), 2.87 (2H, t, *J* 7.6), 6.87 (2H, m), 6.98 (1H, m), 7.13 (3H, m), 7.23 (1H, dd, *J* 2.0, 8.0), 7.34 (1H, td, *J* 2.0, 7.8), 7.42 (1H, d, *J* 2.0), 7.81 (1H, dd, *J* 2.0, 8.0), 9.15 (1H, s, exchange with D_2_O, N*H*), 10.16 (1H, s, exchange with D_2_O, N*H*), 10.32 (1H, s, exchange with D_2_O, N*H*); *δ*_C_ (100 MHz, DMSO-d_6_) 14.5, 21.2, 25.3, 31.5, 108.9, 115.1, 115.5, 117.9, 118.8, 120.0, 120.8, 126.2, 126.8, 128.5, 130.3, 130.4, 133.2, 138.6, 138.7, 139.3, 140.1, 147.1, 168.8, 171.2; *m/z* (ESI negative) 384.0 [M − H]^−^.

### 2′,4′-Difluoro-4-hydroxy-N-(2-oxo-1,2,3,4-tetrahydroquinolin-7-yl)-[1,1′-biphenyl]-3-carboxamide (22)

A solution of **1** (1.0 mmol) was treated with diflunisal (1.0 mmol) in dry *N,N*-Dimethylacetamide (DMA) (4 ml) then *N*-(3-Dimethylaminopropyl)-*N*′-ethylcarbodiimide hydrochloride (EDCI) (2.0 mmol), 1-Hydroxy-7-azabenzotriazole (HOAT) (2.0 mmol), *N,N*-Diisopropylethylamine (DIPEA) (3.0 mmol) were added to reaction mixture. The reaction continued until the consumption of starting materials (TLC monitoring), quenched with 1 M aqueous HCl solution and extracted with ethyl acetate (3 × 15 ml). The combined organic layers were washed with H_2_O (3 × 15 ml), dried over Na_2_SO_4_, filtered, and concentrated to obtain a residue which was purified by silica gel column chromatography eluting with ethyl acetate/*n*-hexane 50% *v*/*v* to afford titled compound.

White solid, yield 15%, m.p.: 281–282 °C; silica gel TLC *R_f_* = 0.23 (Ethyl acetate/*n*-hexane 50% *v/v*); *δ*_H_ (400 MHz, DMSO-d_6_) 2.49 (2H, d, *J* 7.8), 2.89 (2H, t, *J* 7.8), 7.12 (1H, d, *J* 7.6), 7.23 (3H, m), 7.41 (2H, m), 7.65 (2H, m), 8.13 (1H, m), 10.18 (1H, s, exchange with D_2_O, N*H*), 10.47 (1H, s, exchange with D_2_O, N*H*), 12.04 (1H, s, exchange with D_2_O, O*H*); *δ*_C_ (100 MHz, DMSO-d_6_) 25.3, 31.5, 105.4 (t, *J*_C–F_ 26), 108.9, 112.9 (dd, *J*_C–F_ 4, 21), 115.6, 118.5 (d, *J*_C–F_ 19), 120.5, 125.0 (dd, *J*_C–F_ 4, 14), 126.0 (d, *J*_C–F_ 1), 128.7, 130.0 (d, *J*_C–F_ 2), 132.6 (dd, *J*_C–F_ 5, 10), 134.8 (d, *J*_C–F_ 3), 137.9, 139.5, 158.7 (d, *J*_C–F_ 12), 159.1, 161.1 (dd*, J*_C–F_ 3, 12), 163.6 (d, *J*_C–F_ 12), 167.1, 171.2; *δ*_F_ (376 MHz, DMSO-d_6_) −113.8 (1 F, d, *J* 7), −111.5 (1 F, d, *J* 7); *m/z* (ESI negative) 393.0 [M − H]^−^.

### (Z)-4-oxo-4-((2-oxo-1,2,3,4-tetrahydroquinolin-7-yl)amino)but-2-enoic acid (23)

A solution of compound **1** (1.0 mmol) was treated with maleic anhydride (1.05 mmol) in dry DMF then heated up to 150 °C. The reaction continued until the consumption of starting materials, quenched with 1 M aqueous HCl solution to obtain a precipitate which was washed with Et_2_O (3 × 5 ml) and dried under vacuum to obtain desired product.

White solid, yield 30%; m.p.: > 300 °C; *δ*_H_ (400 MHz, DMSO-d_6_) 2.46 (2H, t, *J* 7.6), 2.86 (2H, t, *J* 7.6), 6.67 (1H, d, *J* 15.3), 7.16 (2H, m), 7.23 (1H, dd, *J* 1.8, 8.0), 7.34 (1H, d, *J* 1.8), 10.20 (1H, s, exchange with D_2_O, N*H*), 10.51 (1H, s, exchange with D_2_O, N*H*), 13.03 (1H, s, exchange with D_2_O, O*H*); *δ*_C_ (100 MHz, DMSO-d_6_) 25.2, 31.4, 107.3, 113.9, 120.2, 128.8, 131.4, 138.1, 138.4, 139.5, 162.3, 167.1, 171.1; *m/z* (ESI positive) 261.0 [M + H]^+^.

### N-(2-oxo-1,2,3,4-tetrahydroquinolin-7-yl)methanesulfonamide (24)

Compound **1** (1.2 mmol) was treated with methanesulfonyl chloride (1.01 mmol) in dry THF (3.0 ml) followed by addition of Et_3_N (1.1 mmol). The reaction continued until the consumption of starting materials (TLC monitoring) then quenched with 1 M aqueous HCl solution. Excess of solvents were removed under reduced pressure to obtain a residue which was filtered, washed with Et_2_O (3 × 5 ml) and dried under vacuum to afford titled compound.

White solid, yield 57%; m.p.: 236–237 °C; silica gel TLC *R_f_* = 0.37 (MeOH/DCM 10% *v/v*); *δ*_H_ (400 MHz, DMSO-d_6_) 2.46 (2H, t, *J* 7.6), 2.85 (2H, t, *J* 7.6), 2.98 (3H, s), 6.79 (1H, dd, *J* 2.4, 8.0), 6.84 (1H, d, *J* 2.4), 7.14 (1H, d, *J* 2.4), 9.66 (1H, s, exchange with D_2_O, N*H*), 10.13 (1H, s, exchange with D_2_O, N*H*); *δ*_C_ (100 MHz, DMSO-d_6_) 25.1, 31.4, 39.9, 108.0, 114.5, 120.2, 129.2, 138.2, 139.9, 171.1; *m/z* (ESI negative) 239.0 [M − H]^−^.

### N-(1-methyl-2-oxo-1,2,3,4-tetrahydroquinolin-7-yl)methanesulfonamide (25)

A solution of **24** (0.4 mmol) was treated with iodomethane (0.4 mmol) in dry DMF (3.0 ml) at 0 °C, followed by addition of K_2_CO_3_ (0.4 mmol) then warmed up to rt. The reaction continued until the consumption of starting materials and quenched with slush, acidified with 1 M aqueous HCl solution to obtain a precipitate which was collected, washed with Et_2_O (3 × 5 ml) and dried under vacuum to obtain desired product.

White solid; 80% yield; m.p.: 226–227 °C; silica gel TLC *R_f_* = 0.59 (MeOH/DCM 10% *v/v*); *δ*_H_ (400 MHz, DMSO-d_6_) 2.49 (2H, t, *J* 7.6), 2.90 (2H, t, *J* 7.6), 2.97 (3H, s), 3.22 (3H, s), 6.91 (1H, d, *J* 2.2), 7.01 (1H, dd, *J* 2.2, 8.0), 7.24 (1H, d, *J* 8.0), 10.15 (1H, s, exchange with D_2_O, N*H*); *δ*_C_ (100 MHz, DMSO-d_6_) 25.3, 31.1, 35.8, 38.8, 114.6, 119.9, 123.5, 129.1, 139.7, 141.5, 171.0; *m/z* (ESI positive) 255.0 [M + H]^+^.

### 2,4,6-Trimethyl-1-(2-oxo-1,2,3,4-tetrahydroquinolin-7-yl)pyridin-1-ium perchlorate (26)

A solution of **1** (2.0 mmol) was treated with 2,4,6-trimethylpyrylium tetrafluoroborate (2.4 mmol) in dry methanol (10 ml) then the solution was refluxed for 5 h. Solvent was partially removed, the mixture was cooled down to room temperature and treated with a 1.0 M aqueous solution of HClO_4_ (3.0 equiv.). The precipitate formed was collected by filtration, and crystallised from water to afford the desired product.

Pale yellow solid, yield 40%; m.p.: 280–281 °C; silica gel TLC *R_f_* = 0.10 (MeOH/DCM 10% *v/v*); *δ*_H_ (400 MHz, DMSO-d_6_) 2.37 (6H, s), 2.59 (2H, d, *J* 7.8), 2.63 (3H, s), 3.06 (2H, t, *J* 6.8), 6.97 (1H, d, *J* 2.4), 7.13 (1H, dd, *J* 2.4, 8.0) 7.56 (1H, d, *J* 8.0), 7.94 (2H, s), 10.50 (1H, s, exchange with D_2_O, N*H*); *δ*_C_ (100 MHz, DMSO-d_6_) 22.2, 22.4, 25.5, 30.8, 112.6, 119.8, 127.6, 128.1, 130.9, 138.0, 141.3, 155.6, 159.8, 171.1; *m/z* (ESI positive) 267.0 [M]^+^.

### CA assay

A stopped-flow method^44^ has been used for assaying the CA catalysed CO_2_ hydration activity with Phenol red as an indicator, working at the absorbance maximum of 557 nm, following the initial rates of the CA-catalysed CO_2_ hydration reaction for 10–100 s. For each inhibitor, at least six traces of the initial 5–10% of the reaction have been used for determining the initial velocity. The uncatalysed rates were determined in the same manner and subtracted from the total observed rates. Stock solutions of inhibitor (0.01 mm) were prepared in distilled-deionised water with 5% DMSO and dilutions up to 0.1 nm were done thereafter with the assay buffer. Enzyme and inhibitor were incubated for 6 h^45–48^. The inhibition constant (*K_I_*) was obtained by considering the classical Michaelis–Menten equation which has been fitted by using non-linear least squares with PRISM 3 (La Jolla, CA). All CA isozymes used in the experiments were purified, recombinant proteins obtained as reported earlier by our group^49–59^.

## Results and discussion

### Chemistry

**Scheme 1.**
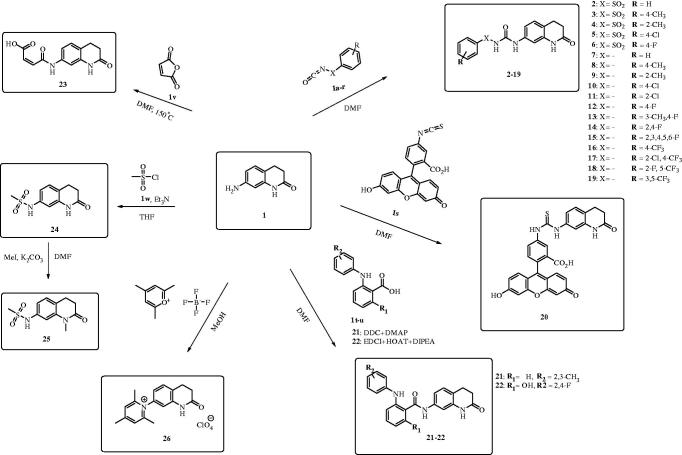
Synthesis of compounds **2–26**.

In a previous report from this group^43^, we showed that 7-amino-3,4-dihydroquinolin-2(1H)-one (**1**) (Scheme 1) possesses interesting CA inhibitory properties against many human isoforms such as hCA VII, IX, XII and XIV, some of which are important drug targets for various applications of the CAIs. The lactam **1** was investigated as a CAI due to its structural similarity with the coumarins, a class of CAIs reported by this group^45–48^. Indeed, unlike other classes of such pharmacological agents, the coumarins act as prodrug inhibitors, being hydrolysed by the CA esterase activity to substituted 2-hydroxy-cinnamic acids, which thereafter bind at the entrance of the active site cavity, far away from the catalytic Zn(II) ion with which most CAIs interact^13^^,^^45^. That region is the most variable among the 15 human CAs, and this explains why coumarins and their derivatives are among the most isoform-selective CAIs reported so far^1^^,^^13^^,^^45–48^. In fact, a large number of substitution patterns at the coumarin ring, isosteric replacements or various other modifications were done on this chemotype, leading to a large number of CAIs possessing interesting properties^13^^,^^45–48^. Thus, the rationale of this work was to derivatise the 7-amino moiety of the lead 1, by reacting it with a variety of agents used earlier for the design of sulfonamide or dithiocarbamate CAIs (Scheme 1)^13–16^^,^^22–25^^,^^35–37^^,^^60^^,^^61^.

As shown in Scheme 1, a multitude of derivatisations of the amino moiety of compound **1** were achieved, such as: (i) reaction with arylsulfonyl isocyanates (leading to arylsulfonylureido derivatives **2–6**); (ii) reaction with isocyanates, leading to ureas **7–19**; (iii) reaction with fluoresceine isothiocyanate, leading to the fluorescent thiourea **20**; (iv) condensation with substituted benzoic acids in the presence of carbodiimides, leading to the amides **21** and **22**; (v) reaction with 2,4,6-trimethyl-pyrylium tetrafluoroborate, leading to the pyridinium salt **26**; (vi) reaction with methylsulfonyl chloride leading to the secondary sulfonamide **24**, which was subsequently methylated with methyl iodide, leading to the 1-*N*-methyl derivative **25**, and (vii) reaction with maleic anhydride leading to the monoamide **23** (Scheme 1). All these compounds were thoroughly characterised by physicochemical procedures which confirmed their structures (see “Materials and methods” for details).

### CA inhibition

Compounds **2–26** were assayed for their CA inhibitory activity by a stopped-flow, CO_2_ hydrase method^44^ against four isoforms of pharmacologic relevance, the cytosolic human (h) hCA I and II, the membrane-anchored hCA IV and the transmembrane, tumour-associated hCA IX (Table 1). The following structure-activity relationship can be observed from the inhibition data of Table 1:

**Table 1.** Inhibition data of hCA I, hCA II, hCA IV, hCA IX with compounds **2–26** reported here and the standard sulfonamide inhibitor acetazolamide (**AAZ**) by a stopped-flow CO_2_ hydrase assay.

	*K_*I*_* (nm)			
Cmp	hCA I	hCA II	hCA IV	hCA IX
**2**	8241.0	7467.6	>10,000	2133.3
**3**	6813.4	6966.7	>10,000	1461.0
**4**	3690.4	6852.2	>10,000	1051.8
**5**	>10,000	6379.1	>10,000	2234.6
**6**	3202.4	4437.9	>10,000	1688.2
**7**	>10,000	>10,000	>10,000	2420.3
**8**	>10,000	>10,000	>10,000	>10,000
**9**	>10,000	>10,000	>10,000	2267.5
**10**	>10,000	>10,000	>10,000	>10,000
**11**	>10,000	>10,000	>10,000	1158.3
**12**	>10,000	>10,000	>10,000	2489.6
**13**	>10,000	>10,000	>10,000	2105.0
**14**	>10,000	>10,000	>10,000	1373.1
**15**	>10,000	7883.8	>10,000	243.6
**16**	>10,000	5724.1	>10,000	>10,000
**17**	5328.9	4973.1	3801.4	2165.2
**18**	8749.6	5490.4	>10,000	1524.5
**19**	>10,000	>10,000	>10,000	2386.7
**20**	>10,000	3378.5	>10,000	1941.1
**21**	>10,000	>10,000	>10,000	>10,000
**22**	>10,000	>10,000	>10,000	2516.7
**23**	>10,000	>10,000	>10,000	1473.3
**24**	>10,000	>10,000	>10,000	292.8
**25**	>10,000	>10,000	>10,000	2758.6
**26**	>10,000	>10,000	>10,000	2658.3
**AAZ**	250	12	74	25

Errors were in the range of ±5–10% of the reported data, from three different assays.

- hCA I was poorly inhibited by most derivatives **2–26**, with only seven of them showing *K_I_*s in the micromolar range (i.e. 3.20–8.75 µm), the remaining ones having *K_I_*s > 10 µm (Table 1). The more effective inhibitors were **2–4, 6, 17** and **18**, which incorporate arylsulfonylureido and ureido moieties. The other substitution patterns led to compounds with much weaker hCA I inhibitory activity.
- hCA II, the dominant cytosolic isoform was generally also poorly inhibited by these derivatives (*K_I_*s > 10 µm) except the arysulfonylureido ones **2–6** (*K_I_*s of 4.43–7.46 µm) the ureas **15–18** (*K_I_*s of 4.97–7.88 µm) and the thiourea **20** (*K_I_* of 3.37 µm), which was the best hCA II inhibitor in the series.
- hCA IV was the least sensitive isoform to these compounds with only one of them (**17**, *K_I_* of 3.80 µm) having an activity <10 µm (Table 1). It is rather difficult to explain this result considering that the inhibition mechanism with these lactams is not yet elucidated.
- The tumour-associated hCA IX was the most inhibited isoform among the four investigated ones, with *K_I_*s ranging between 243.6 and 2758.6 nm (Table 1). Only four derivatives (**8**, **10, 16** and **21**) had *K_I_*s > 10 µm, whereas the best hCA IX inhibitors were **15** and **24** with *K_I_*s of 243.6–292.8 nm. These compounds rather different as the first one is a urea incorporating a pentafluorophenyl moiety, whereas the second one has the secondary sulfonamide functionality. It should be noted that small variations in the structures of such derivatives (as the N1-methylation of **24** leading to **25**) or the reduction of the number of fluorine atoms on the phenyl ring, as in **14**, led to a rather important reduction of the hCA IX inhibitory power compared to **24** and **15**, respectively. Generally, all other arylsulfonylureas/ureas **2–19** (except the two compounds mentioned above as weak inhibitors and **15** which is one of the best) showed a similar behaviour of medium potency hCA IX inhibitors with *K_I_*s of 1.05–2.48 µm.
- All the derivatives reported here showed much weaker CA inhibitory activity compared to the clinically used sulfonamide acetazolamide AAZ (Table 1).

## Conclusions

A series of derivatives was prepared by derivatisation of the 7-amino moiety of 7-amino-3,4-dihydroquinolin-2(1H)-one, a compound investigated earlier as CAI. The derivatisation was achieved by: (i) reaction with arylsulfonyl isocyanates (ii) reaction with aryl isocyanates; (iii) reaction with fluoresceine isothiocyanate; (iv) condensation with substituted benzoic acids in the presence of carbodiimides; (v) reaction with 2,4,6-trimethyl-pyrylium tetrafluoroborate; (vi) reaction with methylsulfonyl chloride and (vii) reaction with maleic anhydride. The new compounds were assayed as inhibitors of four CA human isoforms of pharmacologic relevance, the cytosolic hCA I and II, the membrane-anchored hCA IV and the transmembrane, tumour-associated hCA IX. hCA IX was the most inhibited isoform (*K_I_*s ranging between 243.6 and 2658.3 nm) whereas hCA IV was not inhibited by these compounds. Most derivatives were weak hCA I and II inhibitors, with few of them showing *K_I_*s < 10 µm. Considering that the inhibition mechanism with these lactams is not yet elucidated, exploring a large range of derivatives with various substitution patterns may be useful to identify leads showing isoform selectivity.
